# Healthcare Planning for the Olympics in London: A Qualitative Evaluation

**DOI:** 10.1371/journal.pone.0092338

**Published:** 2014-03-19

**Authors:** Georgia Black, Kostas Kononovas, Jayne Taylor, Rosalind Raine

**Affiliations:** 1 Department of Applied Health Research, University College London, London, United Kingdom; 2 Public Health Specialty Registrar, London Deanery, London, United Kingdom; University of Missouri Kansas CIty School of Medicine, United States of America

## Abstract

**Background:**

Mass gatherings, such as the Olympic and Paralympic Games, represent an enormous logistical challenge for the host city. Health service planners must deliver routine and emergency services and, in recent Games, health legacy initiatives, for the local and visiting population. However there is little evidence to support their planning decisions. We therefore evaluated the strategic health planning programme for the London 2012 Olympic and Paralympic Games to identify generalisable information for future Games.

**Methods:**

We thematically analysed data from stakeholder interviews and documents. The data were prospectively collected in three phases, before, during and after the Games.

**Findings:**

We identified five key themes: (1) *Systemic Improvement* for example in communications, (2) *Effective relationships led to efficiencies and permanent gains*, such as new relationships with the private sector (3) *Difficult relationships led to inefficiencies*, for instance, duplication in testing and exercising emergency scenarios, (4) *Tendency to over-estimate demand* for care, particularly emergency medicine, and (5) *Difficulties establishing a health legacy* due to its deprioritisation and lack of vision by the programme team.

**Interpretation:**

Enduring improvements which are sustained after the Games are possible, such as the establishment of new and productive partnerships. Relationships must be established early on to avoid duplication, delay and unnecessary expense. There should be greater critical evaluation of the likely demand for health services to reduce the wasting of resources. Finally, if a health legacy is planned, then clear definitions and commitment to its measurement is essential.

## Introduction

Mass gatherings, such as the Olympic and Paralympic Games, represent an enormous logistical challenge for the host city. They require the acquisition of new competencies [1] to tackle context-specific and generic issues. For example, local health services must prepare to meet potential demands for emergency care, and address access and transportation difficulties for patients and medical supplies. They must also develop appropriate emergency plans to meet the heightened security risk. Depending on the bid commitments of the host country and local health service structures, health services may need to be provided for athletes and other accredited Olympic representatives (the ‘Olympic Family’).

Little generalisable evidence is available to support health services planning for the Games [2], [3]. The applicability of evidence from other mass gatherings is limited. This is because the Games take place over a long time period (unlike large events such as the World Cup), they involve a mainly young and healthy spectator population (unlike mass gatherings such the Hajj) and they are located over a number of often highly dispersed sites [4]. These factors affect the scale of demand for health and public health services [5].

Evidence from previous Games is limited to specially established medical services provided within Olympic venues for spectators and the ‘Olympic Family’, and to the performance of public health systems, with respect to infectious disease surveillance, control of outbreaks and health promotion [6]–[9]. However the planning required to maintain routine and emergency health services both for the local population and for visitors has not been examined.

Furthermore, there is an emerging interest in the need to demonstrate a sustainable health legacy from the Olympic investment. This has tended to focus on urban regeneration in the areas surrounding the main sporting venues [10]. There is little evidence to inform the establishment of a health legacy [11] nor any high quality research relating to the health impacts of major multi-sport events [12].

We therefore evaluated the planning and delivery of routine and emergency services and of health legacy initiatives during the most recent Games held in London, England in 2012. Our main goal is to present our main findings, focussing on identifying generalisable lessons for health planning and for ensuring a legacy for future Olympics.

## Methods

### Ethics Statement

All participants gave their informed written consent. As the study was considered to be a service evaluation, and participants did not include patients, ethical approval from a committee was not sought. One author (JT) had specific training in informed consent, confidentiality and anonymity as part of her Public Health Speciality qualification; KK and GB had training in research ethics as part of their Masters training. All authors have extensive experience of fully addressing standard ethics guidelines on consent, confidentiality and anonymity, and have produced research protocols, information sheets and consent forms which have been accepted by ethics committees. Participants were recruited from within the programme team and their recommendations for further participants, therefore the identities of participants were known to all taking part. However their anonymised interview transcripts were not available to anyone outside the research team. All raw data including audio files, transcripts and analytic materials such as tables and charts remain restricted to members of the research team. It will not be available to other researchers at any date. Additional consent was taken at each subsequent interview. All approached participants agreed to take part, although some suggested alternative individuals who could contribute further to the evaluation.

### Settings and Participants

#### Interviewing

Interviews were conducted by GB (PhD), KK (MSc) and JT (MSc, MFPH), who were all in a research associate position. JT was also a public health specialty registrar. GB and JT are female, KK is male. All three researchers have significant experience of qualitative interviewing and analysis, including training at a university level. Extra training and support was provided by GB.

A relationship was established with the programme prior to the interviews. Some stakeholders only met the team for the first time at their interview. Participants were informed that the interviewer was from a university, that the evaluation was independent of the programme, and that our primary motivation was to learn some generalisable lessons for future Olympic Games. All interviewers were new to the topic of Olympic health planning and had no prior assumptions about the programme.

We collected qualitative data in three phases, before (Oct–Nov 2011), during (May–July 2012) and after the Games (August–November 2012). We interviewed representatives from all key partner and stakeholder organisations, totalling 56 individuals. NHS London was the public body responsible for planning and delivering routine and emergency services and health legacy initiatives for the local population [13]. We therefore interviewed NHS London’s 2012 public health programme team in addition to Olympic planning leads in NHS primary care clusters (known as primary care trusts, PCTs) and acute hospitals (or hospital groups known as trusts). Using snowball sampling, we recruited additional respondents in partner organisations. These included the London Ambulance Service, the Department of Health (DH), The London Organising Committee of the Olympic and Paralympic Games (LOCOG), Transport for London, the Greater London Authority, the Health Protection Agency and health legacy partners such as GlaxoSmithKline and Sanofi Pasteur Merck Sharp & Dohme (MSD) Limited.

Participants were approached by email for an interview, with a copy of the information sheet attached. Interviews proceeded using open-ended questioning about the programme. Most interviews were conducted at the participant’s place of work, with a small number conducted in public meeting places. The interviews were audio recorded and transcribed, lasting approximately 20–60 minutes. Respondents were interviewed a maximum of three times, depending on, for example, when they joined the programme team (see Table 1). Eighteen respondents were interviewed once, 11 interviewed twice and 27 interviewed all three times. Two interviews were conducted with a group of more than participant, all others were individual. NHS London programme documentation was also analysed, including minutes of programme executive meetings and progress reports.

**Table 1:** Number of participants from each organisation interviewed at each phase of the project.

	*Phase*		
*Organisation*	*I (before Games)*	*II (during Games)*	*III (after Games)*
NHS London	16	14	15
Cluster/borough leads	4	4	4
Department of Health	1	1	3
Olympic (designated) hospitals	3	3	4
Non-designated hospitals	3	3	6
LOCOG	2	3	2
Legacy stakeholders	4	4	4
London Ambulance Service	1	1	1
Other	4	9	7
TOTAL	38	42	39

#### Analysis

Transcriptions of interviews and documents were evaluated using thematic analysis, in accordance with established principles such as inductive line by line coding, thematic grouping of text into codes, revision and verification of coded data, and interpretative, overarching themes [14]. Transcripts were not returned to participants for verification.

The three phases of interviews and the other data sources were analysed independently and concurrently with data collection. This allowed comparisons between datasets to be made. It also enabled us to explore the progression of issues over the course of the programme. Analysis was open-ended, whereby we sought to understand programme components and processes and to identify issues as they arose. Document analysis was undertaken chronologically, to enable exploration of changing priorities and issues over time. Three researchers (GB, KK and JT) open coded different parts of the data using qualitative analysis software (NVivo). Tables of codes and constituent quotations were produced by each researcher on different topics, enabling comparison of issues traced over time. After the third phase of data collection, we brought together our findings from all time periods, and compared themes between different data sources and groups of respondents. We looked for variation within each phase and over time.

The final five themes were decided through discussion and debate with the whole research team, focussing on producing results that were useful for NHS London and future planners. For transparency, each theme is richly described using illustrative quotations from the interviews, provided where needed with information about the participants’ organisation, the stage of planning at which the information was gathered and any other relevant contextual information.

Our research is conducted with a realist perspective. Our findings are likely to be influenced by the context and culture in which they were measured, and we can only present findings from our own perspective. We take it as a principle of qualitative data that in an open ended interview, respondents will mention the topics most important to them. Collecting three types of evidence helped us to triangulate evidence on the phenomenon under study.

## Results

Our full results are published elsewhere [https://www.ucl.ac.uk/dahr/research_pages/index/edit/olympics; 15]. Here, we focus on novel and important findings which have applicability for future Games.

### I. Systemic Improvement

Despite the disappointment relating to the health legacy, respondents reported examples of sustainable systemic improvements that occurred as a result of the Olympics. Arguably, the impact of these system improvements are easier to measure and attribute compared with health improvement impacts, because many changes and adaptations can be put to the test when the NHS is next put under pressure, for example in the busy winter period. NHS London decided to include system improvements as part of their legacy programme, identifying aspects that had been improved in preparation for the Games: “*It’s been partly spotting things in other people’s work that actually they don’t realise is legacy but it is and getting that, not just documented, but getting it recognised […] now actually people will often say their report and then they’ll go oh well of course it’s legacy*.” Improvements were made to mechanisms for overseas charging: “*For example, the overseas charging entitlement work, there’s a good legacy from that in that hopefully everyone will be brought up to speed on the latest regulations and we’ll make sure that the NHS charges more efficiently when overseas.*”

Gains were also identified in other areas: “*I think where you get a legacy that is a natural consequence of running the Olympics, […] we will have much more detailed plans about certain elements of business continuity planning and emergency planning.*”

Systemic improvements in reporting and internal communications were also identified after benefits became apparent during the Games.: *“And we also introduced our own internal conference calls starting at 8 o’clock in the morning. And we’re continuing that, that’s part of our legacy programme. […] it actually pulled all the sites together, all the key decision makers at an operational level.”*

### II. Effective Relationships Led to Efficiencies and Permanent Gains

It was widely reported that the programme successfully delivered due to well-established connections between the NHS London team and effective stakeholders. NHS London were able to work with other parts of the NHS as well as local authorities, private sector partners and groups organising and financing local entertainment and events as part of the cultural Olympiad (i.e. event promoters).

Through their Olympic planning, NHS participants realised that they shared a lot of interests with local authorities and event promoters: “*I’m learning a lot from what [local authorities are] doing and what they’re planning and what their issues are which are very similar to min*e.”

The NHS London programme team also felt that health had become more visible to event promoters and that this would be carried forward in future events: *“we’ve managed to raise the profile of health on event promoters […] they are asking health for information and for support and working with the voluntary sector more for events.”*

Common interests between NHS organisations were also exploited to great effect: *“I work quite closely with the other two designated hospital links. […] we have got to know each other, one of us does something and we send it all round and the others say, “Well, that looks great, and we use it”.*

The private sector also proved to be valuable stakeholders. NHS London admitted that they were surprised at how supportive private sector partners were to work with, and how much funding they could provide: “*That’s a new thing for us in health. There’s not a huge amount of history of working with the corporate sector, and they can put a lot of money into things that we can’t.*”

For their part, the private sector stakeholders enjoyed the relationship and commented on how collaborative and open the NHS were: “*And we’ve often sort of almost second guessed each other, I think, about what we feel needs to happen next […]I think it’s been a very positive relationship.*”

### III. Difficult Relationships Led to Inefficiencies

The relationship between NHS London and LOCOG was acknowledged to be problematic at the start and in need of careful negotiation. At the heart of this relationship was a tension with respect to the position held by LOCOG: a private provider with its own policies and procedures, which overlapped and abutted those of the NHS.

Initially DH, LOCOG, NHS London and the London Ambulance Service found it difficult to work together. Differences in culture and priorities were widely cited, as well as confusion over hierarchy. A member of LOCOG reported: “*it all got very tense and everybody’s vying for position and you know NHS London thought they should be telling me what to do and I should be answering to them.*”

Delays in establishing an effective structure between DH, LOCOG and NHS London led to duplication in effort: *“I think there was a period with a lot of people pretty much doing the same thing and there was a lot of not being very linked together. […] you often went to something and then went to something else and thought, ‘This is exactly the same as we did last week, but there is two people different and all the rest of us are the same.’”*

The most costly sources of duplication were in testing and exercising emergency scenarios, and in the processes of assuring all plans were ready for the Games: *“There was a lot of duplication between the NHS London EP* [emergency planning] *assurance process and then the 2012 team wanting the clusters to do the assurance for the cluster, and then provide the cluster assurance to the 2012 team”*. This duplication was blamed on poor relationships, and a lack of central co-ordination: “*I think an understanding of each organisation about a more coordinated approach to exercises so that we’re not doing another ‘me too’ exercise and testing the same thing is really, really important […] It’s expensive, there are huge cottage industries being set up that aren’t terribly necessary*.”

### IV. Tendency to Over-estimate Demand

In 2011 NHS London established a principle that there should be minimal impact on local health services. However as the start of the Games approached, many respondents asserted that it was better to over-plan than to under-plan. Evidence from previous Olympic and Paralympic Games was often disregarded at this stage on the basis that ‘every Games is different’ and this was used to justify planning for increased demand for services, especially emergency services, in London.

The importance of risk assessment and proportionality was often mentioned by the NHS London team. However, the impending Games, combined with increasing senior management interest, gave rise to growing anxiety about responsibility for Games time delivery among respondents. The scale and political sensitivity of the event dominated participants’ thinking in early interviews. Primary care respondents in particular focussed on the potential scale of major emergency events and the panic that would ensue: “*I think the really interesting challenge, actually, is what I call enhanced business as usual. Because I’m worried about there being an emergency. Clearly…it leaves me cold thinking about what might happen.*”

There was also evidence that NHS London were sensitive to the publicity that the NHS might receive: *“Let’s be absolutely clear that for 62 days we are going to have a gold plated response to this and […] this might feel a little like over-kill [but] our reputation is at stake”*.

Despite this, NHS London tried to promote proportionality through consistent communication about the scale of routine services required: “*Our messages are very clear on that […] we actually are planning for what would be considered a mild winter so that’s what all the data suggest.*”

Yet trusts tended to make their own calculations or to take the upper boundaries of NHS London’s predictions as a starting point, demonstrating their fears: “*We are planning on the top, the estimates we were given was 3–9%, we are planning on the 9%. […] So I have got enough capacity certainly for the 9%, with the two to three admissions a day*.”

Last minute anxiety and the late involvement of senior staff in some organisations led to extreme over-estimation of demand in one case: “*Well, initially, we planned on a 6% increase in […] Accident and Emergency attenders. And then […] the Chief exec, felt we were being too laid back and he said “you’ve got no evidence to support just 6% increase in A&E attenders.” […] And in the end, it was agreed, through the executive board, that we would actually do all our plans based on a 20% increase in A&E attenders*.”

No major incidents were encountered during the Games and the expansion of routine activity was lower than expected. Many respondents expressed disappointment that their plans had not been tested: “*It was all a storm in a teacup over something and nothing*”. Despite this, many respondents justified their approach: “*We’ve spent a lot of money, we’ve got people ready for it but I’d rather have that happen than if something did happen and then the public enquiry starts and we are the ones that are singled out as being not prepared*”. Furthermore, some respondents argued that they had avoided problems due to the comprehensiveness of their planning: *“You over-planned. So nothing happened, why did you bother doing all that planning? Or equally, we did all of that planning and that’s why nothing went wrong. I think it’s probably the latter, that because we did all of the planning and we did all of the engagement and looked at all of the potential issues and we had spent time to look at where the hotspots for travel were and that sort of thing.*”

### V. Difficulties in Establishing a Health Legacy

The London 2012 bid was the first to include a sustainable regeneration plan or legacy. This included a health legacy component, ‘*Go London’*, led by NHS London [16]. The concept of legacy used by NHS London appeared to evolve over the lifetime of the programme, from an initial focus on physical activity and health to a broader concept encapsulating both health and system improvement. A stated aim of the initial strategy was to use what was referred to as the ‘festival effect’ to encourage an Olympics-inspired increase in physical activity (not only in sport participation) across London, especially in socially disadvantaged communities, in order to reduce health inequalities. The final strategy incorporated wider health improvement aims and a specific National Health Service (NHS) focus to use the Olympics to achieve system improvements. Despite these local efforts, as the Games approached, a number of respondents in ‘designated’ hospitals (i.e. designated to provide health care for the Olympic Family) and primary care suggested that the legacy pledge was perhaps the one bid commitment that the NHS could not fulfil. We identified five themes which explain the difficulties they encountered in establishing and demonstrating a legacy:

#### (i) Unclear definition

There was early confusion about whether the health legacy should focus on sport participation, physical activity or broader system improvement. Furthermore, many respondents within the programme team reported difficulties in defining the legacy programme. This was attributed to a lack of clear objectives: *“What I think the programme suffered from was an actual … objective of how it was going to increase physical activity. It did it through one or two campaigns but it never seemed to have a defined structure around how it was going to increase or what impact it was going to have on physical activity levels in London.”* Respondents working in primary care suggested that this was due to lack of conviction about what they themselves wanted, leading to delays: *“It didn’t mean anything to anyone, we left it too late, I think we should’ve launched it but we weren’t clear enough about what we wanted.”*

As the Games drew nearer, the concept of legacy appeared to grow clearer, with a greater drive during this period to label positive Olympic-driven system developments as legacy and focus more attention on legacy initiatives. However, for some respondents the legacy programme remained unclear: “*it’s been quite amorphous, this idea of health legacy. The Go London* [legacy] *programme has been going on since 2009, but there didn’t seem to be a lot of clarity around what the definition of health legacy was*”.

#### (ii) Constriction of the legacy programme

Minutes of the programme executive meetings in 2010 show that DH guidance decreed that funding would not be provided for legacy work. It was decided that finance would be raised using a combination of stakeholder contributions and funds sequestered from other NHS London sources, such as underspend in other areas. In order to raise external funding, NHS London needed to obtain commitments from a substantial number of NHS chief executives as well as the Greater London Authority Mayor’s office.

Respondents from a range of organisations reported that their initial legacy aims were to achieve something ‘*big and exciting’*, with a pan-London focus on increasing physical activity levels. The Olympics inspired grand scale ambitions: *“The legacy […] there was a sense that […] it’s the Olympics – […] it’s got to be something big to be worth doing, for the whole of London.”* NHS London described undertaking a scoping exercise into potential investment to support a programme of this magnitude. This proved to be unfeasible as there was not enough evidence of potential success to garner investment: *“I think we were almost, we had some really good ideas, but we didn’t quite have the evidence base, therefore it never quite cut through with all the partners.”* Several respondents cited the change of government and global recession to be responsible for this: “*I think when the Games were first awarded, then everyone was very excited about what we might do in terms of physical activity schemes and so on. The world was different then, in a pretty major way, the economics were different then*.”

This led to reappraisal and scaling down of the initiatives, with the focus of the programme reduced to supporting a range of local initiatives: *“We started off with these huge ambitions and then obviously the change in the climate politically and the cost cutting exercise meant […] we had to think right, okay, what is important, what do we really need to focus on and needed to narrow it down a lot more.”* The constricted programme was also perceived to exclude the wider population, instead targeting NHS staff: *“I have always felt we were a bit too health service focussed in the way that we were thinking about legacy anyway, […], rather than perhaps looking at the broader audience.”*

#### (iii) Deprioritisation

The primary obstacle to progressing health legacy work in the NHS, as described by all respondents involved in this area, was the ‘deprioritisation’ of the legacy programme in favour of the more pressing work of Olympic operational planning. The consequence of this was that legacy work received a much lower level of funding and resource than other areas. No funding was allocated by the Department of Health for NHS London’s Olympic legacy work as part of the 2012 Programme, although funding was drawn from stakeholders and moved across from other budgets. Central and local perceptions of the consequences of this lack of dedicated funding included delays to initiation of projects while alternative funding sources were being identified. They also experienced difficulties attracting delivery partners without substantial financial resources.

Most respondents reported that the need to ensure service readiness for the Games took precedence over legacy ambitions: *“I would say [legacy] hasn’t been the same focus of my attention, because my greatest focus has been on the NHS having the capability to maintain a safe and timely service through the Olympics.”* Respondents reported that this was partly due to the way the legacy programme was organised in comparison to service planning: *“It would have been nice to have the same sense of urgency and importance of oversight and scrutiny on the legacy work, as compared to the emergency planning, Games time planning. You know, which was extraordinarily military and top down.”*

Practical aspects impacted on legacy programme deprioritisation, such as the low priority in Olympic planning meetings: *“I think the challenge for Legacy, as someone who chairs the meeting that tries to do legacy and planning at the same time, is that it’s always done in the last ten minutes. […] And therefore inevitably to an extent, legacy gets less attention, it doesn’t get the same profile.”* This resulted in it being side-lined: “*the health legacy got knocked into… yeah. Got pushed to the back of the pile*.”

#### (iv) Unsustainability

Some respondents working in hospitals admitted after the Games that their organisations had moved on: “Are we consciously doing anything now with that legacy in this organisation that’s kind of happened or been related? Not at the moment, I don’t think.” This was attributed to the nature of the organisations: “Oh, I think it’s like anything! We’ll all move on, next thing it’s Christmas and it’ll be next year.”

Private sector initiatives also suffered from a lack of planning to safeguard legacy initiatives after the Games: “it was sort of really just for the campaign as it stood, the activities that were planned and there’s nothing planned for the future in that regard.” One private sector respondent identified the lack of funding and re-branding as crucial: “But where I see this now struggling is that I’m not sure how it will continue and who is going to fund it […] it almost needs to take a different form and move away from the Olympics[…] But I’m not sure we’ve got that if I’m honest.”

#### (v) Lack of measurement

No plans were made to formally evaluate legacy initiatives. No baseline, process or outcome measures were identified, defined or recorded, either by NHS London planning team or by the private sector partners: *“Measuring legacy I think is going to be really difficult. I don’t know how they are going to do it.”* Most legacy stakeholders indicated that they would undertake some sort of appraisal, but this would take an anecdotal, post-hoc approach: “*we’re just working out how to collect the feedback. Some of it will be quite anecdotal, because at the moment we don’t have the budget to do a massive study to look at what people thought[…] whether or not they actually have been motivated to change their behaviour in any way and get a bit more active*”. Other respondents mentioned attempts to measure the impact of legacy initiatives, but focused on output rather than outcome measures, such as website hits: *“We’ve found a sample way of measuring how much we’ve increased referrals onto walking groups and things like that. […] we’ve got the website, obviously we’ve got the number of hits on that […] also we’ve got the tracker, there is a tracker device in it that people can then go on to register for, and monitor their own activity.”*

## Discussion

This, the first independent evaluation of the planning and delivery of local and emergency services during the Olympic and Paralympic Games has identified five main themes with particular applicability for future Olympic hosts. Our results suggest that if a health legacy is envisaged, then greater attention to its definition, prioritisation and measurement is crucial. We have also highlighted the enduring system improvements which are likely to be established in health settings where attention and resources are focussed on procedures such as emergency preparedness and business continuity planning. Significant financial and network gains are possible when effective relationships are established early in the process, but poor relationships between health organisations produce duplication, delay and expense. Finally, we have illustrated the difficulties in proportionate planning for the Games.

Our results indicate that partnership working between the healthcare and other public and private bodies bring public health knowledge and important resources into the health service. The private sector can also offer organisational quality and service improvement expertise to publicly funded organisations [20]. It is important to identify such benefits and their drivers, because significant barriers to public-private partnerships and threats to public sector culture have been identified elsewhere [21].

The difficult relationships between the Olympic organising committee, the government and health providers were not unique to London. Indeed this has been shown to be a problem at many recent Olympics and could be improved by early agreement on co-operative roles and governance [22], [23]. Relationship problems were eventually overcome in London, but still resulted in expensive and unnecessary duplication.

It is understandable that concerns about potential disaster at the Games over-rode evidence-based models on likely levels of demand for emergency and routine healthcare. This has been demonstrated elsewhere, notably with respect to heightened security measures [24]. If over-planning is to be avoided or reduced, improved estimates of demand during mass events is required, as well as effective communication about confidence in these estimates [25].

Future hosts attempting to institute a health legacy must maintain focus on both the vision and the detail if it is to be effective. Evidence of a health legacy is not only scarce, but hard to demonstrate [17]. It requires evidence of changes over time which are associated with the Olympic health initiatives. Formal, adequately resourced evaluations therefore need to be embedded at the inception of legacy planning to enable long term benefits for the host population to be examined [3], with evidenced legacy measurement methodology [18]. Without robust, long term evaluations, major multi-sport events should not be justified in terms of benefits to the host population [12]. Well-conducted legacy and system improvement programmes could have the greatest gains in countries that are resource poor, where substantial quality improvement is needed in readiness for the Games [19].

A key strength of our research methods (prospective qualitative data collected at pertinent points over time) was that we captured multiple perspectives, and identified processes and priorities as they evolved and changed. Our research design was efficient, feasible and replicable in other mass gathering contexts. However we recommend that future evaluations also include the collection of quantitative data and detailed information on demand for healthcare in all settings during the Games. Such data would be invaluable to future Games planners.

## Conclusions

The Games present a significant challenge to planners of routine and emergency health services within the host city. This paper has presented generalisable findings relating to partner engagement, financial planning, mass gatherings event pressures, and the difficulties of demonstrating legacy effects. We have demonstrated the value and feasibility of using qualitative research methods to capture evidence which can improve both the efficiency of routine health service provision during such events and the success of legacy initiatives.
